# Heritable and Nonheritable Rumen Bacteria Are Associated with Different Characters of Lactation Performance of Dairy Cows

**DOI:** 10.1128/msystems.00422-22

**Published:** 2022-09-14

**Authors:** Xin-Wei Zang, Hui-Zeng Sun, Ming-Yuan Xue, Zhe Zhang, Graham Plastow, Tianfu Yang, Le Luo Guan, Jian-Xin Liu

**Affiliations:** a Institute of Diary Science, College of Animal Sciences, Zhejiang University, Hangzhou, China; b Institute of Genetics and Reproduction, College of Animal Sciences, Zhejiang University, Hangzhou, China; c Department of Agricultural, Food and Nutritional Science, University of Alberta, Edmonton, Canada; Colorado State University

**Keywords:** heritable microbial taxa, microbiability, lactation traits, dairy cows

## Abstract

Recent studies have reported that some rumen microbes are heritable. However, it is necessary to clarify the functions and specific contributions of the heritable rumen microbes to cattle phenotypes (microbiability) in comparison with those that are nonheritable. This study aimed to identify the distribution and predicted functions of heritable and nonheritable bacterial taxa at species level in the rumen of dairy cows and their respective contributions to energy-corrected milk yield, protein content and yield, and fat content and yield in milk. Thirty-two heritable and 674 nonheritable bacterial taxa were identified at species level, and the functional analysis revealed that predicted microbial functions for both groups were mainly enriched for energy, amino acid, and ribonucleotide metabolism. The mean microbiability (to reflect a single taxon’s contribution) of heritable bacteria was found to range from 0.16% to 0.33% for the different milk traits, whereas the range for nonheritable bacteria was 0.03% to 0.06%. These findings suggest a strong contribution by host genetics in shaping the rumen microbiota, which contribute significantly to milk production traits. Therefore, there is an opportunity to further improve milk production traits through attention to host genetics and the interaction with the rumen microbiota.

**IMPORTANCE** Rumen bacteria produce volatile fatty acids which exert a far-reaching influence on hepatic metabolism, mammary gland metabolism, and animal production. In the current study, 32 heritable and 674 nonheritable bacterial taxa at species level were identified, and shown to have different microbiability (overall community contribution) and mean microbiability (the average of a single taxon’s contribution) for lactation performance. The predicted functions of heritable and nonheritable bacterial taxa also differed, suggesting that targeted nutritional and genetic breeding approaches could be used to manipulate them to improve dairy cow performance.

## INTRODUCTION

Rumen microbes degrade cellulose, hemicellulose, and pectin in plants, and subsequently produce volatile fatty acids (VFAs) that account for 60% to 70% of the metabolizable energy for the host animal (1). Increasing numbers of studies have uncovered the relationships between rumen microbiota and production traits of dairy and beef cattle such as methane emission (2, 3), feed efficiency (4, 5), and milk production and quality (6, 7). Hence, manipulation of rumen microbiota can be one of the potential strategies to optimize production performance in cattle.

Despite the known dietary effect on the richness and diversity of rumen microbiota (8), increasing evidence has shown that host factors (breed, genetic variation, etc.) can affect the composition and function of the rumen microbiome in cattle. Some rumen microbial taxa have been shown to be heritable (2, 9–11) and some of these heritable taxa are associated with single nucleotide polymorphisms (SNPs) in both dairy cows (3) and beef cattle (5). In beef cattle, 59 of 174 rumen microbial taxa (at various taxonomic levels) were found to be heritable with an estimated narrow sense heritability (h^2^) ≥ 0.15; and 19 SNPs were associated with 14 of them (5). Similarly, 39 genera of the core rumen microbiota were reported to be heritable with h^2^ ≥ 0.2 (3), and 6% of bacterial taxa (eight of 144 bacterial genera) at the operational taxonomic unit (OTU) level were found to be heritable (h^2^ > 0) in dairy cows (2). From these studies, Sasson et al. (10) described the relationship between heritable microbiota and host phenotypes (milk protein [MP] and milk fat [MF], etc.) at species level OTU, and compared the mean correlation of heritable and overall microbes to host traits, showing that on average, heritable microbes show a higher correlation to host traits. Wallace et al. (3) reported the explainability (predictive ability) of core microbiota based on ridge regression for different host traits, and compared the mean explanatory (prediction) power of heritable and nonheritable core microbes in explaining host traits and experimental variables, revealing that heritable microbes showed higher explainability of host traits and experimental variables. These results suggest further comparison of the contribution to the host phenotypic traits of the heritable and nonheritable microbes in microbial communities (or subcommunities) would be worthwhile. Those microbial communities making a high phenotypic contribution may provide new options for phenotypic improvement.

In recent years, the concept of “microbiability” has been applied to estimate the contribution of gastrointestinal tract microbiota to the host phenotype (12). In dairy cattle, rumen microbial composition can explain 15% microbiability for the amounts of acetone and β-hydroxybutyric acid (two metabolites related to ketosis in dairy cows) in milk (13, 14). In addition to the microbiability of health-related traits, recent studies have also estimated rumen microbiability of rumen microbial composition, functions, and metabolites for milk protein yield (MPY) as 17.8%, 21.6%, and 29.8%, respectively (15). MPY is a novel trait that can reflect both milk and protein yield with some cows having high MPY (high in both milk yield [MY] and MP) that has advanced the traditional dairy cow production traits that were focused only on MY, MP, and MF (16, 17). Although our previous study identified a significant difference in the rumen microbiota between high and low MPY cows (17), it did not assess the heritability of the rumen bacteria and whether the heritable and nonheritable bacteria taxa contribute to MPY traits similarly or differently.

We hypothesized that heritable rumen bacteria have different functions from nonheritable ones, and the contribution of heritable rumen microbes to MPY is higher than that of nonheritable microbes. Therefore, the objectives of this study were to identify the heritable and nonheritable bacteria at species level based on 16S rRNA gene sequencing and host genotyping, to investigate the functional difference of rumen bacteria, and to determine the microbiability of heritable and nonheritable groups to milk production traits with a focus on MPY. The analyses of the bacterial taxa in the current study were conducted based on the species level as it provides a more direct linkage of their functional contributions to the host production traits.

## RESULTS

### Identification of heritable and nonheritable taxa in the rumen of dairy cows.

**FIG 1:**
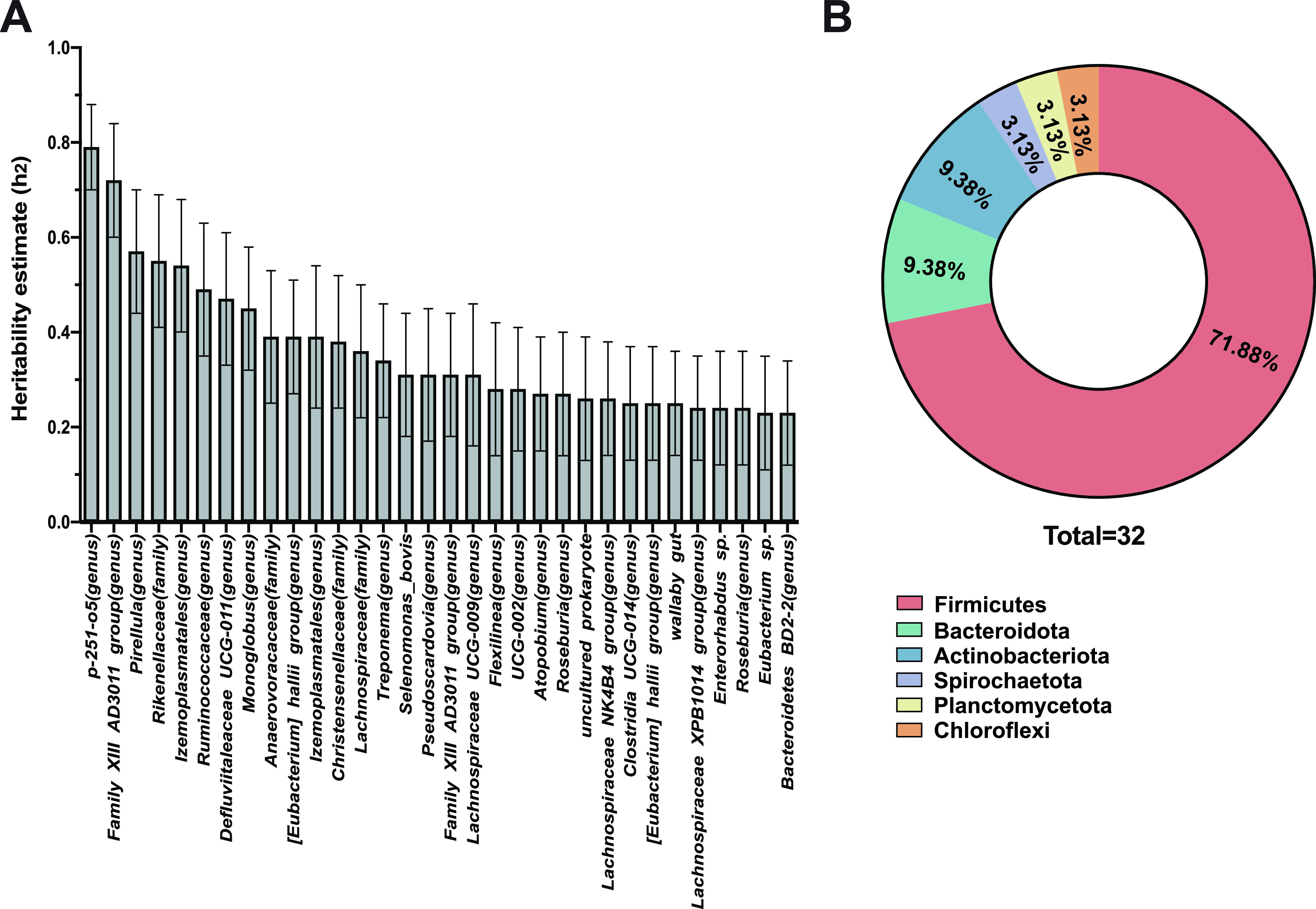
Narrow sense heritability analysis based on the genetic relatedness matrix (GRM). (A) Heritability estimated results. *x* axis: Name of the microbes correlating with the animal genotype; *y* axis: Heritability estimate (h^2^; bar plots show mean estimate per microbe). Error bars stand for standard error of mean. (B) The distribution of heritable bacteria among different phyla.

We analyzed SNP genotypes of 398 Holstein dairy cows and obtained rumen bacterial profiles of 361 dairy cows using amplicon sequencing. Genotyping was performed using Bovine Geneseek Genomic Profiler Low-Density 31K Beadchip (30,108 SNPs) for 285 dairy cows and GGP Bovine 100K Beadchip (99,229 SNPs) for 113 dairy cows. A total of 361 dairy cows passed quality control of both microbiota (with quality score higher than 20) and genotyping (with minimum allele frequency higher than 5% and genotyping call rate greater than 90%). After genotype imputation and quality control, 87,056 SNPs were used to construct the genetic relatedness matrix G (Fig. S1). Population structure analysis did not indicate any genetic difference between the groups of animals (Fig. S2). A total of 18,351 amplicon sequence variants (ASVs) were identified with a mean sequence depth of 56,781 ± 558 (mean ± SE [standard error of mean]) reads per sample (Table S1) with the sequence depth ranging from 25,155 to 85,366. In total, 35 phyla were identified with Firmicutes (49.3 ± 0.4%, mean ± SE) and Bacteroidota (38.8 ± 0.6%, mean ± SE) being the most abundant phyla (Table S2). At the genus level, 814 taxa were identified with 185 unclassified and 629 classified (Table S2), of which *Prevotella* (22.0 ± 0.6%, mean ± SE) and *Rikenellaceae_RC9_gut_group* (4.5 ± 0.08%, mean ± SE) were the most predominant genera. At the species level, 1,705 taxa were identified with 1,139 unclassified and 566 classified (Table S2), of which the unclassified species belonging to the genus of *Prevotella* (8.2 ± 0.1%, mean ± SE) and the family of *Lachnospiraceae* (5.0 ± 0.1%, mean ± SE) the most abundant species. With taxa found in at least 20% of the cows, 706 bacterial taxa at species level (500 were unclassified accounting for 77.8 ± 0.3% relative abundance and 206 were classified accounting for 20.7 ± 0.4% relative abundance) were selected to construct the microbiota relationship matrix M (Fig. S1) for downstream analysis. Significant correlation (with *R* = 0.42, *P* value = 1E-04) was identified between the genetic relatedness matrix G and the microbiota relationship matrix M. From them, 32 heritable (with h^2^/SE greater than 2 and *P* value less than 0.05 were considered significantly heritable) and 674 were classified as nonheritable (without significant h^2^) bacteria at the species level (Table S3). Among the heritable microbes, 32 taxa had h^2^ > 0.2, including 8 highly (h^2^ > 0.4) and 24 moderately heritable bacteria (0.2 < h^2^ < 0.4) (Fig. 1A). The 32 heritable bacteria belonged to six phyla, including Firmicutes (18), Bacteroidota (3), Actinobacteriota (3), Spirochaetota (1), Planctomycetota (1), and Chloroflexi (1) (Fig. 1B). Some species level taxa belonging to Firmicutes phylum had high estimated h^2^, including species of the unclassified genus of *Family_XIII_AD3011_group* (h^2^ = 0.72 ± 0.12; mean ± SE), the unclassified genus of *Izemoplasmcoreatales* (h^2^ = 0.54 ± 0.14; mean ± SE), and the unclassified genus of *Ruminococcaceae* (h^2^ = 0.49 ± 0.14; mean ± SE). In addition, most (two of three) heritable species level taxa belonging to Bacteroidota phylum had high h^2^ estimation, such as species of the unclassified genus of *p-251-o5* (h^2^ = 0.79 ± 0.09; mean ± SE) and unclassified family of *Rikenellaceae* (h^2^ = 0.55 ± 0.14; mean ± SE).

10.1128/msystems.00422-22.1 FIG S1 The genetic relationship matrix (G) plot among dairy cows were constructed by genome information (A). Download FIG S1, TIF file, 2.6 MB. Copyright © 2022 Zang et al. 2022 Zang et al. https://creativecommons.org/licenses/by/4.0/ This content is distributed under the terms of the Creative Commons Attribution 4.0 International license.

10.1128/msystems.00422-22.2 FIG S2 Principal-coordinate analysis (PCoA) based on PLINK in relation to subcorhort. Download FIG S2, EPS file, 0.9 MB. Copyright © 2022 Zang et al. 2022 Zang et al. https://creativecommons.org/licenses/by/4.0/ This content is distributed under the terms of the Creative Commons Attribution 4.0 International license.

10.1128/msystems.00422-22.4 TABLE S1 Sequence depth of each sample. Download Table S1, XLSX file, 0.02 MB. Copyright © 2022 Zang et al. 2022 Zang et al. https://creativecommons.org/licenses/by/4.0/ This content is distributed under the terms of the Creative Commons Attribution 4.0 International license.

10.1128/msystems.00422-22.5 TABLE S2 Taxa relative abundance at phylum, family, genus, and species level results with 16S rRNA sequencing in SILVA 138 database. Download Table S2, XLSX file, 0.1 MB. Copyright © 2022 Zang et al. 2022 Zang et al. https://creativecommons.org/licenses/by/4.0/ This content is distributed under the terms of the Creative Commons Attribution 4.0 International license.

10.1128/msystems.00422-22.6 TABLE S3 Heritability estimate results at species level. Download Table S3, XLSX file, 0.04 MB. Copyright © 2022 Zang et al. 2022 Zang et al. https://creativecommons.org/licenses/by/4.0/ This content is distributed under the terms of the Creative Commons Attribution 4.0 International license.

### Host SNPs associated with ruminal heritable taxa.

Using genome-wide association study (GWAS) analysis, a total of 63 SNPs were significantly (*P_adj_* < 0.05) associated with 12 heritable bacterial species level taxa that belonged to Firmicutes, Bacteroidota, and Actinobacteriota phyla. These SNPs were located on 20 of the 28 Bos taurus autosomes (BTA) (excluding BTA 10, 11, 15, 16, 20, 22, 24 and 25) (Table 1; Fig. S3). Among 63 SNPs, 40 of them were significantly associated with species level taxa belonged to Firmicutes, such as ARS-BFGL-NGS-1550 on BTA 5 (*P_adj_* = 2.26E-03), BovineHD0100031775 on BTA 1 (*P_adj_* = 9.95E-03), and BTB-01139875 on BTA 8 (*P_adj_* = 2.95E-05) (Table 1). The SNP-associated species level taxa included unclassified genera of *[Eubacterium]_hallii_group*, *Family_XIII_AD3011_group*, *p-251-o5*, *Lachnospiraceae_XPB1014_group*, *Pseudoscardovia*, *Roseburia*, *Izemoplasmatales*; unclassified family of *Rikenellaceae*, *Lachnospiraceae*; and *wallaby_gut*. (Table 1).

**TABLE 1:** Identified bovine SNPs associated with rumen microbial taxa

SNPs	Position	Alleles	Gene	Consequence	Associated taxon	*P value*	*FDR* ^*a*^	Milk traits
ARS-BFGL-NGS-1550	5:104506754	A/G	ANO2	intron_variant	*Family_XIII_AD3011_group(genus)*	2.60E-08	2.26E-03	MY, MFY
BovineHD0100031775	1:111405342	A/G	KCNAB1	intron_variant	*wallaby_gut*	3.43E-07	9.95E-03	
ARS-BFGL-NGS-77195	14:12352611	G/A	NC region^*b*^	intergenic_variant	*Rikenellaceae(family)*	1.90E-08	1.39E-03	
BTB-01532239	14:22781305	A/G	XKR4	intron_variant	*p-251-o5(genus)*	8.29E-10	7.22E-05	
ARS-BFGL-NGS-8960	23:14416017	G/A	LRFN2	intron_variant	*p-251-o5(genus)*	3.56E-08	1.03E-03	
ARS-BFGL-NGS-89152	17:1094949	A/G	NC region^*b*^	intergenic_variant	*p-251-o5(genus)*	2.65E-08	1.03E-03	
BTB-01139875	8:77583129	A/G	SLC28A3	intron_variant	*Lachnospiraceae(family)*	3.39E-10	2.95E-05	
BovineHD0800011917	8:39967424	G/A	SLC1A1	intron_variant	*Lachnospiraceae(family)*	2.08E-09	9.04E-05	
BovineHD0800006915	8:23068842	G/A	NC region^*b*^	intron_variant	*Lachnospiraceae(family)*	1.52E-08	3.32E-04	
BovineHD1400007028	14:22579809	A/C	NC region^*b*^	intergenic_variant	*Lachnospiraceae(family)*	1.15E-08	3.32E-04	
ARS-BFGL-NGS-55148	2:64906276	A/G	LYPD1	intron_variant	*Lachnospiraceae(family)*	2.57E-08	4.47E-04	
ARS-BFGL-BAC-34984	21:17364351	G/A	AGBL1	intron_variant	*Lachnospiraceae(family)*	6.79E-08	9.86E-04	
BovineHD0600030451	6:115930865	G/A	SH3BP2	intron_variant	*Lachnospiraceae(family)*	9.47E-08	1.18E-03	
Hapmap40935-BTA-27343	18:61476153	G/A	NC region^*b*^	intron_variant	*Lachnospiraceae(family)*	1.53E-07	1.66E-03	
BovineHD0200019138	2:65813784	G/A	NC region^*b*^	intergenic_variant	*Lachnospiraceae(family)*	5.02E-07	4.37E-03	
ARS-BFGL-NGS-23697	21:62001635	A/G	NC region^*b*^	intergenic_variant	*Lachnospiraceae(family)*	5.67E-07	4.49E-03	
BovineHD0600010892	6:38259537	C/A	NC region^*b*^	intergenic_variant	*Lachnospiraceae(family)*	7.29E-07	5.29E-03	
BovineHD2100005615	21:19225035	A/G	NTRK3	intron_variant	*Lachnospiraceae(family)*	9.58E-07	5.56E-03	
BovineHD2700013341	27:41896909	A/G	THRB	downstream_gene_variant	*Lachnospiraceae(family)*	9.48E-07	5.56E-03	
BovineHD1800018300	18:62895629	G/A	CDC42EP5	upstream_gene_variant	*Lachnospiraceae(family)*	8.97E-07	5.56E-03	
BovineHD0600009841	6:33920158	G/A	CCSER1	intron_variant	*Lachnospiraceae(family)*	1.28E-06	6.97E-03	
chr28_26919160	28:26756476	G/A	PALD1	intron_variant	*Lachnospiraceae(family)*	1.46E-06	7.50E-03	
ARS-BFGL-NGS-23408	12:67949031	G/A	GPC6	intron_variant	*Lachnospiraceae(family)*	1.75E-06	8.10E-03	
ARS-BFGL-NGS-95596	28:19968429	G/A	NC region^*b*^	intergenic_variant	*Lachnospiraceae(family)*	1.77E-06	8.10E-03	
ARS-BFGL-NGS-33852	2:65726104	C/A	NC region^*b*^	intergenic_variant	*Lachnospiraceae(family)*	2.08E-06	8.89E-03	
ARS-BFGL-NGS-82610	27:41883327	G/A	THRB	intron_variant	*Lachnospiraceae(family)*	2.14E-06	8.89E-03	
BovineHD0300004801	3:14784950	G/A	ARHGEF2	upstream_gene_variant	*Lachnospiraceae(family)*	2.41E-06	9.21E-03	MP
BovineHD1700014837	17:50488654	C/A	TMEM132B	intron_variant	*Lachnospiraceae(family)*	2.50E-06	9.21E-03	
BovineHD0200020270	2:70187252	G/A	NC region^*b*^	intergenic_variant	*Lachnospiraceae(family)*	2.54E-06	9.21E-03	
BovineHD0600024595	6:87896899	G/A	NC region^*b*^	intergenic_variant	*Lachnospiraceae_XPB1014_group(genus)*	5.31E-08	1.17E-03	
BTB-00265951	6:88893733	A/G	CXCL5	upstream_gene_variant	*Lachnospiraceae_XPB1014_group(genus)*	5.31E-08	1.17E-03	
BTA-31690-no-rs	1:61189193	G/A	NC region^*b*^	downstream_gene_variant	*Lachnospiraceae_XPB1014_group(genus)*	2.10E-08	1.17E-03	
BovineHD0200009949	2:33743321	A/G	NC region^*b*^	intergenic_variant	*Lachnospiraceae_XPB1014_group(genus)*	5.36E-08	1.17E-03	
BovineHD1200016394	12:59463519	G/A	NC region^*b*^	intergenic_variant	*Pseudoscardovia(genus)*	9.94E-14	8.65E-09	
BTB-00740910	19:17720890	G/A	PSMD11	intron_variant	*Pseudoscardovia(genus)*	6.26E-12	2.73E-07	
ARS-BFGL-NGS-21636	12:25233090	A/G	NC region^*b*^	intron_variant	*Pseudoscardovia(genus)*	4.05E-10	1.18E-05	
BovineHD1900005868	19:19945005	A/G	SLC13A2	intron_variant	*Pseudoscardovia(genus)*	1.20E-09	2.62E-05	
ARS-BFGL-BAC-6557	1:1954528	C/A	SON	3_prime_UTR_variant	*Pseudoscardovia(genus)*	7.25E-09	1.26E-04	
BTA-122671-no-rs	14:79800919	G/A	NC region^*b*^	intergenic_variant	*Pseudoscardovia(genus)*	9.95E-09	1.44E-04	
BTA-23872-no-rs	12:50367458	A/G	TBC1D4	intron_variant	*Pseudoscardovia(genus)*	3.60E-08	4.48E-04	MF
Hapmap47254-BTA-33890	13:76564375	G/A	NC region^*b*^	intergenic_variant	*Pseudoscardovia(genus)*	1.27E-07	1.38E-03	
BovineHD0900000055	9:263529	A/G	NC region^*b*^	intergenic_variant	*Pseudoscardovia(genus)*	2.95E-07	2.56E-03	
BovineHD0300010345	3:33090696	G/A	RBM15	intron_variant	*Pseudoscardovia(genus)*	2.77E-07	2.56E-03	
BovineHD0600011201	6:39832870	A/C	SLIT2	intron_variant	*Pseudoscardovia(genus)*	4.98E-07	3.61E-03	MP
BTB-00250899	6:39885647	G/A	SLIT2	intron_variant	*Pseudoscardovia(genus)*	4.98E-07	3.61E-03	MP
ARS-BFGL-NGS-33300	13:74690747	A/G	PCIF1	intron_variant	*Pseudoscardovia(genus)*	7.05E-07	3.83E-03	
BovineHD1300021786	13:74704084	G/A	ZNF335	intron_variant	*Pseudoscardovia(genus)*	7.05E-07	3.83E-03	
ARS-BFGL-NGS-30910	13:74713520	A/G	ZNF335	intron_variant	*Pseudoscardovia(genus)*	7.05E-07	3.83E-03	
BovineHD0800007957	8:26305056	A/G	ADAMTSL1	intron_variant	*Pseudoscardovia(genus)*	6.93E-07	3.83E-03	
Hapmap51817-BTA-22015	26:2874049	G/A	NC region^*b*^	intergenic_variant	*Pseudoscardovia(genus)*	9.30E-07	4.50E-03	
ARS-BFGL-NGS-72624	12:35202030	A/G	NC region^*b*^	downstream_gene_variant	*Pseudoscardovia(genus)*	9.06E-07	4.50E-03	
BovineHD0700032588	7:109037932	A/G	LOC1NC	non_coding_transcript_exon_variant	*Pseudoscardovia(genus)*	1.59E-06	6.91E-03	
BovineHD0800007957	8:26305056	A/G	ADAMTSL1	intron_variant	*Defluviitaleaceae_UCG-011(genus)*	5.33E-09	4.64E-04	
BTB-01286589	8:21227745	G/A	NC region^*b*^	intergenic_variant	*Defluviitaleaceae_UCG-011(genus)*	9.35E-08	2.71E-03	
Hapmap47254-BTA-33890	13:76564375	G/A	NC region^*b*^	intergenic_variant	*Defluviitaleaceae_UCG-011(genus)*	3.80E-07	6.61E-03	
DB-271-seq-rs109067623	5:22483919	A/G	NC region^*b*^	intergenic_variant	*Defluviitaleaceae_UCG-011(genus)*	3.60E-07	6.61E-03	
ARS-BFGL-NGS-55148	2:64906276	A/G	LYPD1	intron_variant	*Roseburia(genus)*	1.50E-08	1.31E-03	
BovineHD0600024595	6:87896899	G/A	NC region^*b*^	intergenic_variant	*Roseburia(genus)*	8.41E-08	2.44E-03	
BTB-00265951	6:88893733	A/G	CXCL5	upstream_gene_variant	*Roseburia(genus)*	8.41E-08	2.44E-03	
BTB-01721944	4:44456308	A/G	NAPEPLD	intron_variant	*Roseburia(genus)*	4.63E-08	2.02E-03	
BTA-122414-no-rs	4:34823772	A/G	LOC1NC	intron_variant	*Roseburia(genus)*	3.53E-08	2.02E-03	
BovineHD0500035128	5:118732607	A/G	NC region^*b*^	intergenic_variant	*[Eubacterium]_hallii_group(genus)*	1.30E-08	1.13E-03	
BovineHD1300009423	13:31909385	A/G	NC region^*b*^	intergenic_variant	*Izemoplasmatales(genus)*	7.07E-08	6.15E-03	

a For each microbial taxonomic feature, *P* value was adjusted into genome-wide false discovery rates (*FDRs*) using the Benjamini-Hochberg method. Associations with *FDR *< 0.01 were considered significant, and associations with 0.01 < *FDR *< 0.05 were regarded as suggestively significant.

b NC region, noncoding region.

10.1128/msystems.00422-22.3 FIG S3 Manhattan and Q-Q plot of single nucleotide polymorphism loci for heritable rumen bacteria. Download FIG S3, TIF file, 1.1 MB. Copyright © 2022 Zang et al. 2022 Zang et al. https://creativecommons.org/licenses/by/4.0/ This content is distributed under the terms of the Creative Commons Attribution 4.0 International license.

### Relationship between heritable and nonheritable bacteria and core and noncore bacteriome.

**FIG 2:**
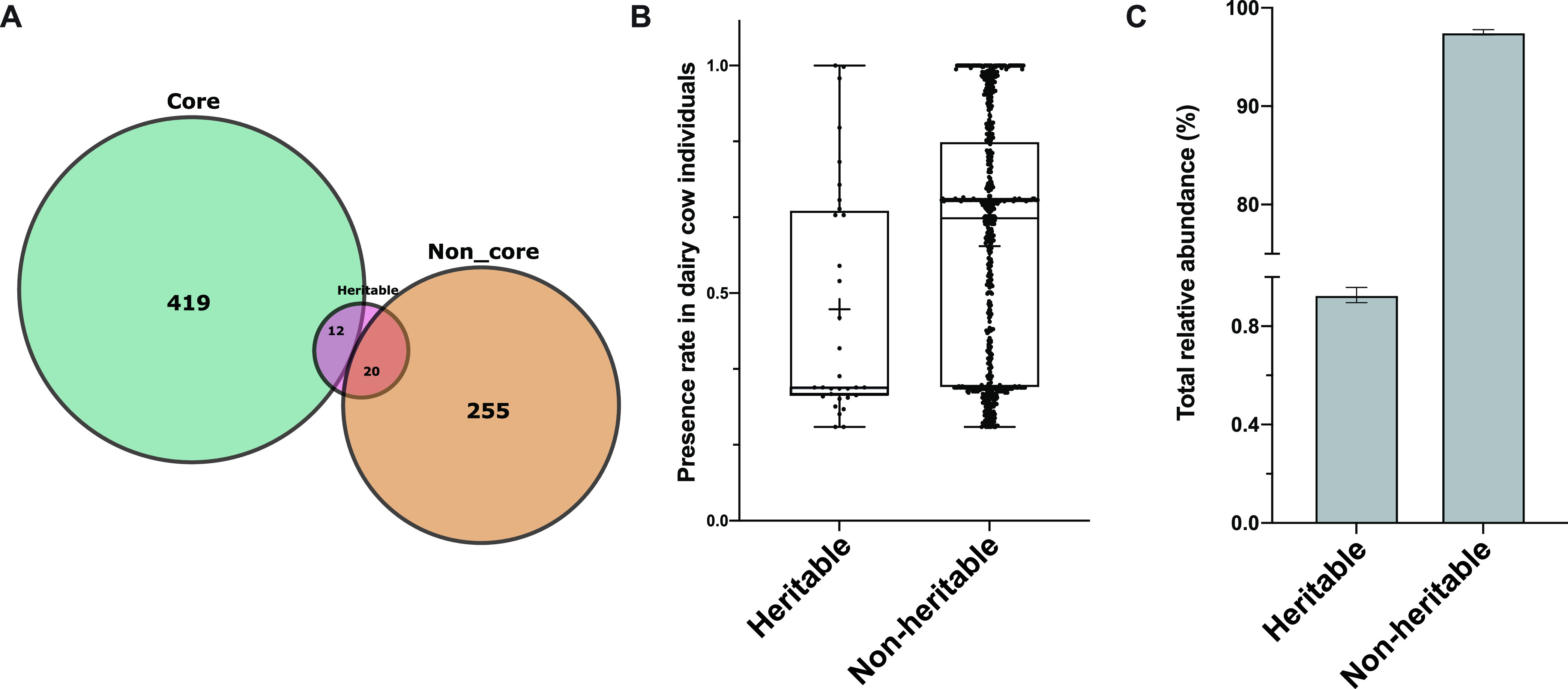
Relationships among core, noncore bacteria and heritable, nonheritable bacteria. (A) The distribution of heritable bacteria between core and noncore bacteria. (B) Presence rate of heritable and nonheritable bacteria in individual dairy cows. (C) Total relative abundance of heritable bacteria. Error bars stand for standard error of mean.

We further defined taxa present (with relative abundance >0) in at least 50% of animals as core bacteria and those present in 20% to 50% of animals as noncore bacteria (3). Among the 706 detected species level bacterial taxa, 431 were defined as the core bacteriome, and 275 were defined as the noncore bacteriome (Fig. 2A). The dominant core bacterial phyla were Firmicutes (47.4%), Bacteroidotas (23.8%), and Proteobacterias (7.8%), and each of the remaining 11 minor phyla accounted for <4.0% of abundance (Table S4). The dominant noncore bacterial phyla were Bacteroidotas (8.5%) and Firmicutes (5.7%) (Table S5). Among the 32 species level heritable bacteria, 12 species level taxa were core bacteria and 20 species level taxa were noncore bacteria (Fig. 2A). The prevalence of heritable and nonheritable bacteria differed in the studied cow population with average presence rate of 46.46% for heritable bacteria and of 60.32% for nonheritable bacteria (Fig. 2B). The relative abundance of the heritable and nonheritable bacteria was 0.93% and 97.52%, respectively (Fig. 2C).

10.1128/msystems.00422-22.7 TABLE S4 The relative abundance of core bacteria at species level. Download Table S4, XLSX file, 1.9 MB. Copyright © 2022 Zang et al. 2022 Zang et al. https://creativecommons.org/licenses/by/4.0/ This content is distributed under the terms of the Creative Commons Attribution 4.0 International license.

10.1128/msystems.00422-22.8 TABLE S5 The relative abundance of noncore bacteria at species level. Download Table S5, XLSX file, 0.7 MB. Copyright © 2022 Zang et al. 2022 Zang et al. https://creativecommons.org/licenses/by/4.0/ This content is distributed under the terms of the Creative Commons Attribution 4.0 International license.

### Microbiability of heritable and nonheritable bacteria and lactation traits.

**FIG 3:**
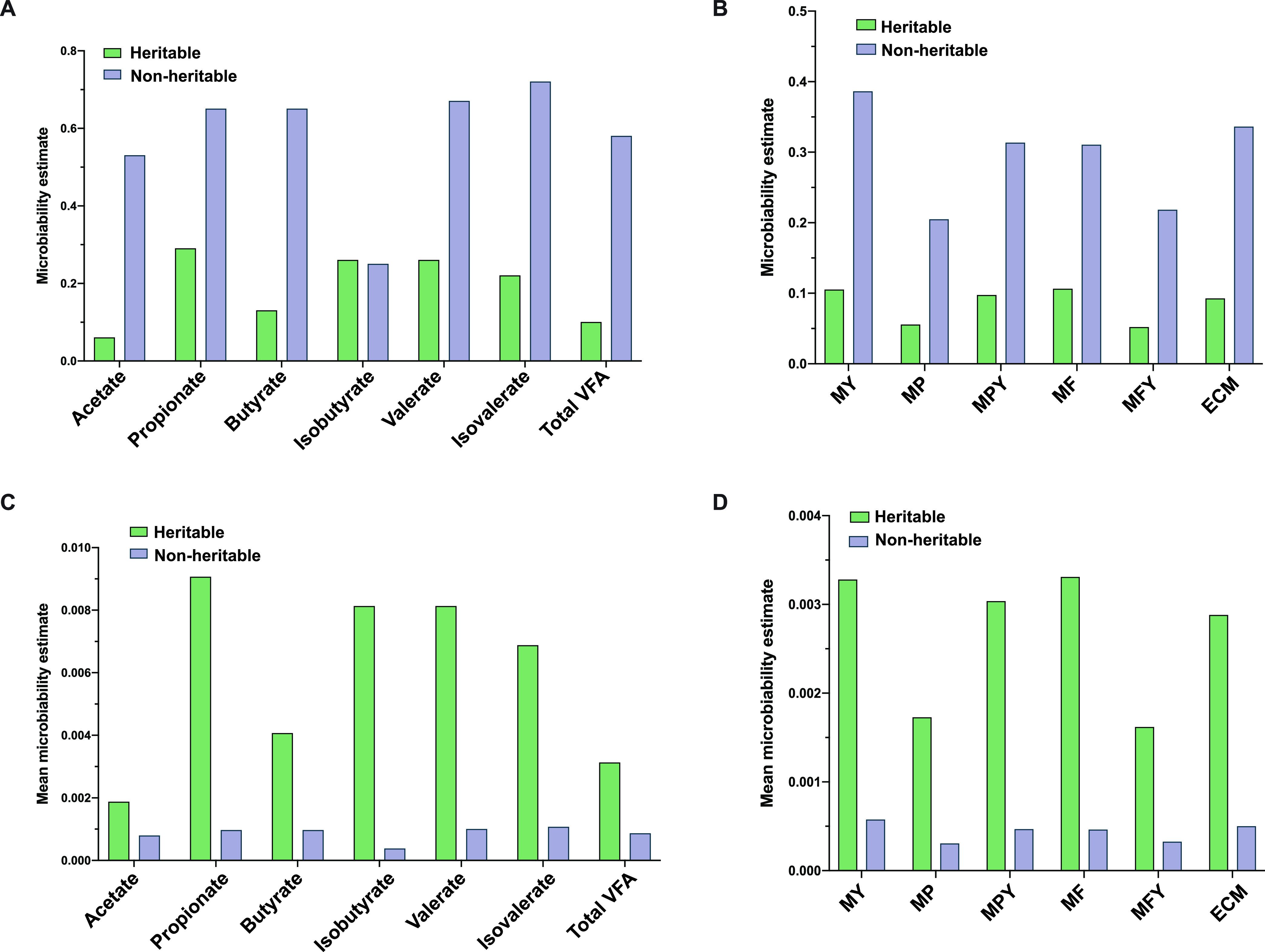
The microbiability estimation for different milk traits and volatile fatty acids with heritable microbiota and nonheritable microbiota using ASReml software. The *x* axis represents the heritable and nonheritable microbiota, and the *y* axis represents the average value and SE value of microbiability. (A) and (B) Total microbiability estimation for milk traits and volatile fatty acids. (C) and (D) Mean microbiability estimation for milk traits and volatile fatty acids. SE, standard error of mean.

To identify to what extent heritable and nonheritable rumen bacteria could affect the production of VFAs and lactation traits, we estimated microbiability for each phenotype separately. The microbiability of heritable and nonheritable bacteria for acetate, propionate, butyrate, isobutyrate, valerate, isovalerate, and total VFA were six and 53%, 29 and 65%, 13 and 65%, 26 and 25%, 26 and 67%, 22 and 72%, and 10 and 58%, respectively (Fig. 3A). The microbiability of heritable and nonheritable microbiota to MY, energy-corrected milk yield (ECM), MP, MPY, MF, and milk fat yield (MFY) were 11 and 39%, six and 20%, 10 and 31%, 11 and 31%, five and 22%, and nine and 34%, respectively (Fig. 3B). We also calculated the average contribution of heritable and nonheritable bacteria, defined as mean microbiability (see details in Materials and Methods). The mean microbiability of heritable and nonheritable bacteria for acetate, propionate, butyrate, isobutyrate, valerate, isovalerate, and total VFA was 0.19 and 0.08%, 0.91 and 0.10%, 0.41 and 0.10%, 0.81 and 0.04%, 0.81 and 0.10%, 0.68 and 0.11%, and 0.31 and 0.09%, respectively (Fig. 3C). The mean microbiability of heritable and nonheritable bacteria in terms of variation for MY, ECM, MP, MPY, MF, and MFY was 0.33 and 0.06%, 0.29 and 0.05%, 0.17 and 0.03%, 0.30 and 0.05%, 0.33 and 0.05%, and 0.16 and 0.03%, respectively (Fig. 3D). Heritable bacteria had a greater mean contribution than nonheritable bacteria for rumen VFAs and lactation performance. However, this group had a lower overall contribution (microbiability) for most of the individual rumen VFAs (except for isobutyrate) and all lactation traits.

### Predicted functions of heritable and nonheritable bacteria and relationship between heritable bacteria and lactation traits.

**FIG 4:**
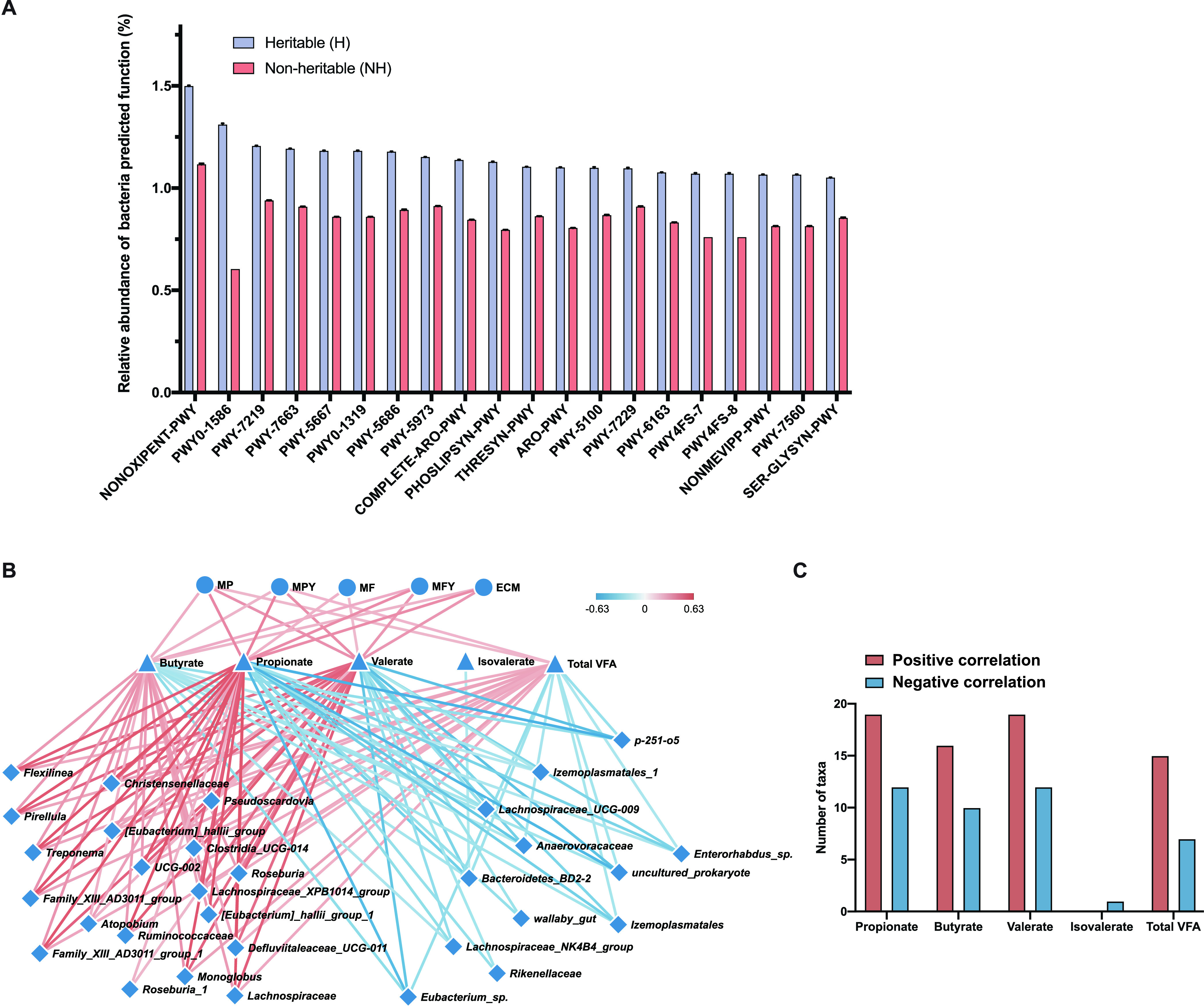
Relative abundance of predicted functions of heritable and nonheritable bacteria and relationship between heritable bacteria and lactation traits. (A) The relative abundance of functions for heritable and nonheritable bacteria. (B and C) Correlation among heritable bacteria volatile fatty acids and milk lactation traits, respectively.

In total, 243 and 374 predicted biochemical functions were obtained for heritable and nonheritable bacteria, respectively. To investigate the major functional differences between heritable and nonheritable bacteria, the top 20 most abundant predicted functions were selected and compared. The top 20 most abundant predicted functions included amino acid metabolism, such as superpathway of aromatic amino acid biosynthesis, superpathway of l-threonine biosynthesis, superpathway of l-serine and glycine biosynthesis I, as well as fatty acid metabolism (gondoate biosynthesis, chorismate biosynthesis I) (Fig. 4A). Compared with nonheritable bacteria, predicted functions for heritable bacteria were mostly enriched for anabolism (Fig. 4A).

Further Spearman correlation analysis between the lactation performance and VFAs showed propionate and valerate were positively associated with MY (*P*_adj_ < 0.05), and VFAs were positively associated with lactation performance (*P*_adj_ < 0.05). We then assessed the correlations between the VFAs and heritable bacteria and found 19, 16, 19, and 15 heritable bacteria were positively associated with propionate, butyrate, valerate, and total VFA, respectively. Twelve, 10, 12, one, and seven heritable bacteria were negatively correlated with propionate, butyrate, valerate, isovalerate, and total VFA, respectively (Fig. 4B and C). In addition, when correlations between heritable rumen bacteria and lactation performance were assessed, 18, 16, 18, 18, and 18 heritable bacteria each were found to be positively associated with MP, MPY, MF, MFY, and ECM. Thirteen, 13, 10, 11, and 12 heritable bacteria were negatively correlated with MP, MPY, MF, MFY, and ECM, respectively (Table S6).

10.1128/msystems.00422-22.9 TABLE S6 The correlations results between heritable rumen bacteria and lactation performance. Download Table S6, XLSX file, 0.02 MB. Copyright © 2022 Zang et al. 2022 Zang et al. https://creativecommons.org/licenses/by/4.0/ This content is distributed under the terms of the Creative Commons Attribution 4.0 International license.

## DISCUSSION

Recent studies have shown that some rumen microbes are heritable with high h^2^ (h^2^ > 0.4) in dairy cows (2, 3, 10, 11) and beef cattle (5, 9). From our study, five heritable bacterial taxa (belonging to genera of *Roseburia*, Treponema, families of *Lachnospiraceae*, *Spirochaetaceae*, and *Christensenellaceae*) were identified as similar to previous studies (3, 5), suggesting that some of heritable bacteria are core rumen microbiota present in the rumen of both dairy cows and beef cattle due to their adaptive colonization and key functions in rumen fermentation. However, some of the heritable taxa with high h^2^ were only found in our study, such as *Pirellula*, *Izemoplasmatales*, and *Monoglobus* genera. Differences in cattle breeds among studies (Holstein dairy cows in our study) versus purebred Angus and Charolais beef cattle (5) and Holstein-Friesian dairy cattle in previous studies (3, 10), may affect the profiles of heritable bacteria and their estimated heritability. The estimated h^2^ significantly differs between beef and dairy cattle. For example, 75% of heritable species level taxa had moderate h^2^ (0.2 < h^2^ < 0.4) estimation in the current study, which is consistent with the findings in dairy cows by Wallace et al., who found 87% of heritable species level taxa had moderate h^2^ (0.2 < h^2^ < 0.4) estimation (3). However, our results were different from findings in beef cattle in which these taxa had low h^2^ (h^2^ < 0.2) (5), suggesting that selection for different production purposes in cattle can shape the type of heritable rumen bacteria. It is known that many factors can affect the microbiota, and these factors may interact with each other. For example, ruminal pH is one of the factors that can affect the rumen bacteriome. However, such a cofactor was not well defined in previous studies. Unlike previous studies, we included pH as a cofactor (random effect) in the GWAS and heritability models to minimize its effect (649 of 706 rumen bacteria can be affected by pH factor in our study) on the relative abundance of rumen bacteria and enhance the accuracy of heritability estimation. Therefore, the identified heritable taxa are likely more affected by the genotype of the cows included here compared to previous studies.

Previous studies identified that some rumen microbiota were heritable (2, 3, 5, 9–11). Our current study used ASVs-based taxonomic classification instead of OTUs compared with the previous dairy and beef cattle studies. ASVs-based method can detect small biological sequence variants and discard technical errors introduced by library preparation and sequencing technology. This increases the taxonomical resolution of the results. Our study identified 32 heritable bacteria, which is higher than those reported by Sasson et al. (10), who reported 13 heritable bacterial OTUs, and less than those reported by Wallace et al. (3) with 39 heritable bacteria. It has been reported the lactation stage can influence the rumen bacteria composition (2); thus, the different lactation stages among studies could partially contribute to the variation in the number of heritable bacterial species detected. In this study, the cows were in midlactation and days-in-milk ranging from 125 to 185 versus whole lactation stage and days-in-milk ranging from 81 to 240 in Wallace et al. (3), and early to midlactation stage and days-in-milk ranging from 50 to 150 in Sasson et al. (10). Additionally, previous studies had identified heritable microbiota at OTU, species, and genus levels (2, 3, 5, 9–11). Compared with the findings at species level reported by Wallace et al. (3) (six of 39 were heritable), a similar number of heritable bacteria were classified at species level and were heritable (five of 32) in our study. Some studies focused on heritable bacteria at genus level (2, 5), but classification of the heritable bacteria at species level is essential to understand their functions and impact on cattle production traits. It is well known that different species belonging to the same genus have varied functions, which can impact the rumen microbial fermentation outcomes significantly. Although we used the species level taxa for the analysis, many of them were unclassified species. Therefore, future studies of heritable bacteria at species or strain level based on metagenomics and/or culturomics are needed to provide a better understanding of their functions and to precisely determine the function of heritable bacteria in the rumen and whether they can serve as bacterial markers for the genetic selection.

Recently, the role of rumen bacteria in milk production traits and VFAs has been increasingly recognized (7, 19). Previous microbiability studies estimated rumen microbiota microbiability to cattle methane performance (2) which have focused on the contribution of the overall microbiota to host traits (12, 20) but did not evaluate the difference between heritable and nonheritable taxa. We estimated the microbiability (contribution) of heritable and nonheritable bacterial taxa based on a mixed linear model. Wallace et al. (3) assessed the explainability (similar to the microbiability in our study with a different estimation method) of MY, which is lower than in the current study (0.2 in Wallace et al. versus 0.39 in current study). The potential reason for the difference in microbial contributions to the milk production traits between the two studies is the use of different models, microbial taxa included, and cattle populations (size and breed). However, microbiability does not reflect the average single taxon contribution. Thus, we proposed the concept of “mean microbiability” to represent the contribution of a single taxon. We have a basic assumption when calculating the microbiability, that is, each taxon obeys the same distribution (variance): N (0, σ^2^), where σ^2^ refers to the variation of the bacteria population. Mean microbiability implies the high value of a single taxon’s contribution to the performance traits. Therefore, we estimated microbiability and mean microbiability to reflect the overall community and a single taxon’s average contribution to lactation performance traits, respectively. In the current study, the heritable and nonheritable microbiota had different mean microbiability for MPY with a higher contribution from heritable bacteria than nonheritable bacteria, suggesting more important roles of heritable bacteria in affecting dairy cow lactation performance.

As one of the identified novel milk production traits, MPY was related to specific heritable bacteria, such as species of the unclassified family of *Lachnospiraceae*, *Ruminococcaceae*, and *Christensenellaceae*. Consistent with the previous study in which these taxa were reported to be associated with MY (7), it suggests that these heritable bacteria can be potentially selected by host genetics and they could be considered microbial targets to improve MY. Another study also found that the rumen unclassified family of *Ruminococcaceae* was associated with rumen fermentation measures (e.g., acetate, propionate) (3), and our study found some taxa (e.g., species of the unclassified family *Ruminococcaceae*, *Lachnospiraceae*) have significant correlation with propionate. These findings suggest that heritable rumen bacteria may contribute more to producing propionate, the main precursor for liver glycogenesis that directly affects lactation performance. In addition to heritable bacteria in this study, we identified some nonheritable (potentially regulated by nongenetic factors) rumen bacteria (e.g., *Anaerovorax*, *Tyzzerella*), and some of them (e.g., *Moryella*, *Fibrobacter*) can negatively impact MY (7). A previous study showed that the abundance of *Anaerovorax* differed significantly between different diets (control diet, control diet plus rumensin, and control diet plus rumensin and 4% fat) (21). Although the cows were fed the same diet and raised in the same farm for each cohort, the variation in intake and the diet/environment may be different. Future studies are needed to assess how the diet and management can impact the abundance of nonheritable bacteria. Moreover, a recent study has revealed that core and noncore microbiota in the rumen both play vital roles in affecting lactation performance (7). Similar results were observed in our study, both core bacteria, and noncore bacteria, were significantly associated with rumen VFAs (species of the unclassified family *Ruminococcaceae* related with propionate) and 12 of core bacteria were heritable and 20 of noncore bacteria were heritable. This has advanced our knowledge showing that both heritable and nonheritable bacteria can be members of core and pan rumen microbiota (individualized rumen microbiota); however, it is unclear whether they may interact with each other to affect the milk production traits. Future studies to evaluate such observations in the rumen of different ruminant species is needed to validate these findings.

Furthermore, we found that the heritable and nonheritable bacteria have different potentials. The functions of heritable bacteria were mainly enriched in fatty acid, amino acid, and energy metabolism, while nonheritable bacteria are mainly enriched in amino acid and ribonucleotide metabolism. Fatty acid metabolism was enriched in heritable bacteria, suggesting that heritable bacteria play important roles in carbohydrate metabolism which is the fundamental step to convert plant materials to VFAs in the rumen. Higher concentrations of VFAs can provide more energy for dairy cows and promote the production performance of dairy cows (22), suggesting that genetic selection of these microbes may enhance VFAs metabolism in the dairy cows. For the energy metabolism function, heritable rumen bacteria such as *Lachnospiraceae* (most abundant family of Firmicutes) can ferment starch and other sugars to produce butyrate and other short-chain fatty acids (23), which are then essential for host energy metabolism. Butyrate can serve as energy substrates for the host. Fatty acid oxidation metabolizes butyrate to CO_2_ or acetyl-CoA for lipid and glucose synthesis (18). Indeed, functional enrichment analysis revealed the pyruvate metabolism function is associated with the heritable bacteria. Rumen bacteria use glycolysis to generate pyruvate, one of the precursors for hepatic gluconeogenesis, to produce glucose (24), which is then used to synthesize lactose (the determinant of MY) in the mammary gland of dairy cows (25). As heritable microbes are directly affected by host genetics, their relationship with host energy metabolism further highlights the future implications for their use as selection makers for cattle breeding.

In addition to energy metabolism, another factor that contributes to milk production traits is amino acid metabolism. Both heritable and nonheritable bacteria had enriched predicted functions of amino acid metabolism with two and six of them enriched for heritable and nonheritable bacteria, respectively. Studies have shown that the addition of 2-hydroxy-4-(methylthio)-butanoic acid (HMB) and l-lysine-HCl to diets with inadequate methionine and lysine resulted in the increased MY and MPY (26). Liu et al. (27) have found that *Ruminococcaceae* (heritable family detected in this study) was positively associated with l-Glutamate and l-Valine in the rumen fluid, suggesting that the heritable *Ruminococcaceae* enriched in the rumen through genetic selection in the future can improve rumen nitrogen metabolism for better lactation performance. Future studies on the functions of heritable and nonheritable bacteria through metagenome, metabolome and culture-based studies are required to confirm the functional differences.

In addition to identifying that both heritable and nonheritable bacteria are associated with host metabolism and can influence lactation performance, this study further revealed that the host genotype could simultaneously affect the rumen microbiota and milk production. We found that the SNP: BovineHD0600009841 located in coiled-coil serine rich protein 1 (*CCSER1*) gene was associated with the bacterial species belonging to the unclassified family of *Lachnospiraceae*. *CCSER1* was reported to be involved in the cell division defect with a potential impact on microbiome components in human colorectal cancer (28). The expression of this gene has been found in rumen epithelial tissues of dairy and beef cattle based on their transcriptome profiles (29, 30), suggesting that this SNP may potentially influence the rumen microbiota by affecting the cell functions of the rumen epithelial wall. Of the heritable bacteria-associated SNPs, five are also associated with the variation of lactation performance. For example, the SNP: BovineHD0300004801 (associated with variation in the abundance of the unclassified family of *Lachnospiraceae*) is located within the gene Rho/Rac Guanine Nucleotide Exchange Factor 2 (ARHGEF2) on BTA 3. Although the function of the gene has not been well studied in cattle, it has been reported to be involved in epithelial barrier permeability in human (31). We speculate that this gene may also have similar functions in the rumen epithelia that can affect barrier function which has been reported to affect host-microbial interactions in the rumen (32). The SNP: BovineHD0300004801 (associated with unclassified family of *Lachnospiraceae*) was significantly associated with MP (33). Meanwhile, SNPs on BTA 12 *(BTA-23872-no-rs* [associated with unclassified genus of *Pseudoscardovia*]) overlap with quantitative trait loci (QTLs) for MF; and BTA 5 *(ARS-BFGL-NGS-1550* [associated with unclassified genus of *Family_XIII_AD3011_group*]) overlap with QTLs for MY and MFY (33). Besides, these SNP were associated with heritable rumen species level taxa (unclassified genus of *Family_XIII_AD3011_group*, *Pseudoscardovia*, and unclassified family of *Lachnospiraceae*) were positively correlated with the proportion of propionate. It is reported that intraluminal infusion of propionate could significantly improve MY and MPY (34). These results suggest these QTLs may have pleiotropic effects on both rumen microbiota and lactation performance and may partly explain the contribution of milk production traits by the rumen microbiome. Identifying heritable bacteria associated SNPs suggests that such association can be passed to offspring and can be used to genetically select cows with these bacteria in the rumen and better milk production performance for the cows, which warrants future studies.

### Conclusion.

In this study, we identified 32 heritable bacterial species level taxa in the rumen of dairy cows, and that they have diverse distribution between core and noncore bacteria. These taxa enriched amino acid and energy metabolism functions and had a larger mean microbiability to the lactation traits compared with nonheritable microbiota. Heritable bacteria such as unclassified species from genera of *Roseburia* and *Ruminococcaceae* were significantly associated with rumen fermentation, thus affecting milk production traits in dairy cows. Our results provide a comprehensive assessment of the contribution of heritable and nonheritable microbiota, and provide novel insights into a microbe-host genetics interaction relationship. Understanding the contributions of heritable and nonheritable bacteria to milk production traits will provide a scientific foundation to develop targeted and precision manipulation strategies of rumen microbial groups to improve rumen functions and cattle performance. Heritability and microbiability estimation provide the basis to potentially select or breed cattle for better rumen microbial functions where heritable microbes positively affect and contribute to performance.

## MATERIALS AND METHODS

### Animal study, sample collection, and phenotypic measurement.

Animal care and experimental procedures were approved by the Animal Care Committee of Zhejiang University (Hangzhou, China). All the experimental procedures followed the rules and guidelines of Zhejiang University. Our study consisted of 398 midlactation Holstein dairy cows from a commercial dairy farm (days-in-milk: 155 ± 30.2, parity: 2.61 ± 1.18, mean ± standard deviation) with 285 animals sampled in 2016 and 113 animals sampled in 2019. The feeding and management of the experimental cows have been described previously (16). In brief, the animals were fed three times daily at 0630, 1400, and 2000 h at a forage-to-concentrate ratio of 43:57, and had free access to water. They were reared under the same farm environment and management with daily cleaning of manure. The MY was recorded for three consecutive days, and milk samples were collected at the ratio of 4: 3: 3 in the morning (0630 h), afternoon (1400 h), and evening (2000 h) through the pipeline milk system. The MP and MF were measured using infrared analysis with a spectrophotometer (Foss-4000; Foss Electric A/S, Hillerød, Denmark), and MPY and MFY were the results of MP and MF multiplied by MY, respectively. ECM was calculated by the following equation (35): ECM (kg/d) = 0.3246 × MY (kg/d) + 13.86 × MFY (kg/d) + 7.04 × MPY (kg/d).

Rumen fluid was collected using an oral stomach tube before morning feeding, as described previously (36). The pH value of rumen fluid was measured with a portable pH instrument (FE-28, China) immediately after collection. The rumen fluid was stored at −80°C and used for DNA extraction and rumen bacterial profiling. Blood samples were collected at the same time when the rumen fluid sample was collected. Ten mL of blood samples were collected from jugular venous blood into tubes containing heparin sodium. After collection, the tubes were gently shaken upside-down 8 to 10 times to prevent blood aggregation, then stored at −80°C before DNA extraction for genotyping.

### 16S rRNA gene sequencing, data processing, and functional analysis.

In total, rumen fluid of 361 cows was collected from two animal cohorts (2016 and 2019) and subjected to microbial profiling using amplicon sequencing. Specifically, for the 2016 cohort (*n* = 254), total DNA extraction and 16S rRNA gene amplicon sequencing methods have been described previously (7). Briefly, total DNA extracted from each rumen fluid sample was amplified using 341F/806R primer set (37) (5′-CCTAYGGGRBGCASCAG-3′/5′-GGACTACNNGGGTATCTAAT-3′); PCR solution contained 0.5 U of *Taq* polymerase (TransGen Biotech, Beijing, China), 25 μL of 10 × PCR buffer, 200 μM each dNTP, 0.2 μM each primer, and 2 μL of DNA (50 ng/μL). The amplicon was sequenced on an Illumina (San Diego, CA) HiSeq platform using the pair-ended 2 × 250 bp at Novogene Bioinformatics Technology Co. Ltd. (Beijing, China). For the 2019 cohort (*n* = 107), total DNA was extracted from rumen fluid samples using the FastDNA Spin Kit for Soil (MP Biomedicals, USCAT NO.116560–200, Omega Bio-tek, Norcross, GA, USA), and the genomic DNA were amplified using the 515F/806R primer set (5’-GTGY-CAGCMGCCGCGGTAA-3′/5′-GGACTACNVGGGTWTCTAAT-3′) with a PCR thermocycler (ABI, CA, USA), and the hypervariable region V3-V4 of the bacterial 16S rRNA gene were amplified with primer pairs. The amplicon was sequenced on an Illumina MiSeq platform (Illumina, San Diego, USA) using the pair ended 2 × 300 bp at Majorbio Bio-Pharm Technology Co. Ltd. (Shanghai, China).

To ameliorate potential batch effects in the data set, samples were pooled for the DADA2 pipeline to resolve singleton amplicon sequence variants (38). Sequence demultiplexing and quality control was performed using QIIME2 v2.0.6 (39) (https://view.qiime2.org) with q2-demux plug-in to demultiplex the original sequence data and data set 2 for denoising. Data with a quality score higher than 20 were used for downstream analysis (40). All amplicon sequence variants were mapped to the latest SILVA138 database (June 2020 release) to obtain a taxonomic classification at species level. Only taxa found in at least 20% of the cows were considered detected taxa and included in the downstream analysis (7). In addition, we defined taxa present (with relative abundance >0) in at least 50% of animals as core bacteria, and those present in 20% to 50% of animals were defined as noncore bacteria (3).

The microbial data processed by QIIME 2 were used as input data to predict microbial functions using PICRUSt2 v2.1.0–b (41) (https://github.com/picrust/picrust2/wiki). Microbial functional analysis was performed separately for the heritable and nonheritable microbiome, and top 20 enriched predicted functions were selected for downstream analysis of the heritable and nonheritable bacteria.

### Genotyping and genotype imputation analysis.

DNA was extracted from whole blood according to Wizard Genomic DNA purification kit (https://www.promega.jp/resources/protocols/technical-manuals/0/wizard-genomic-dna-purification-kit-protocol/) and subjected to genotyping at Delta Genomics (Edmonton, AB, Canada). QuantiFluor dsDNA system (Promega, Madison, WI) was used for quantitative analysis of DNA samples. Genotyping was performed using Bovine Geneseek Genomic Profiler Low-Density 31K Beadchip for 285 dairy cows and GGP Bovine 100K Beadchip (Neogen Inc, Lincoln, NE) according to the Illumina Infinium Ultra manual (Illumina, San Diego, CA) for 113 dairy cows. Illumina HiScan was used to scan the chip, and Genome Studio V2.0 software (https://support.illumina.com/array/array_software/genomestudio/downloads.html) was applied to process and export the original data according to the selection criteria of at least 90% animal call rate. A total of 31,000 to 100,000 imputations were executed by FImpute (Version 2.2) and Beagle (Version 3.3.2) with default parameter settings and last got 97,228 SNPs. Quality control of SNPs was carried out with PLINK V1.07 (42) according to the following criteria: (i) minimum allele frequency >5%; and (ii) genotyping call rate >90%. Population structure analysis was performed with PLINK V1.07.

### Estimation of narrow sense heritability and microbiability of rumen bacteria.

The h^2^ of rumen bacteria was estimated using the restricted maximum likelihood method in ASReml V4.1 software (43). The h^2^ analysis assumes the data follow a normal distribution (44, 45), and the log_10_-transformed data have been applied in many studies (2, 5, 46) to transform microbiota data for improving the normality of microbiota data. Therefore, the taxa found in at least 20% of the cows (5) were log_10_-transformed and used to calculate h^2^ (47). A total of 361 animals with complete data of microbial abundance, genotype was used for downstream analysis (Table S7). To capture the additive genetic relationships among individuals, the genomic relationship matrix (G) was constructed based on the SNPs after quality control using the method previously developed in the Synbreed software package in R (48). The following linear model was used to correct the fixed effects on microbial taxa, i.e., the relative abundance of bacteria (49, 50):

(1) y=Xb+e

10.1128/msystems.00422-22.10 TABLE S7 Lactation traits of 398 mid-lactation dairy cows. Download Table S7, XLSX file, 0.03 MB. Copyright © 2022 Zang et al. 2022 Zang et al. https://creativecommons.org/licenses/by/4.0/ This content is distributed under the terms of the Creative Commons Attribution 4.0 International license.

Where ***y*** is microbial taxa; ***b*** is the fixed effect vector, including overall mean and the effects of subcohort, parity, age, days-in-milk and pH; ***X*** is the design matrix; ***e*** is residual error.

Using the corrected microbial phenotype and genetic relatedness matrix G, the phenotypic variance that is due to additive genetic effect was calculated in the following animal model:

(2) $$y^{\text{*}}=1\mu +\mathrm{Za}+e$$

Where y^*^ is the corrected microbial feature in previous calculation; ***μ*** is the overall mean and 1 is a column vector with ones; ***a*** is the random additive genetic effect following a distribution of ***N***(0, **G**σ_*a*^2^_), with the genomic relationship matrix *G* and the additive genetic variance σ_a_^2^; ***Z*** is the design matrix; ***e*** is the random residual effect following **N**(0, **I**σ_e^2^_) with identity matrix I and residual variance σ_e^2^_. The following equation was used to calculate h^2^ (43):

(3) $$h^{2}={\text{σ}}_{{\text{a}}^{2}}/({\text{σ}}_{{\text{a}}^{2}}+\sigma_{{\text{e}}^{2}})$$

The bacteria with h^2^ estimation significantly greater than zero ($\frac{h^{2}}{\mathrm{SE}}>2$, *P* < 0.05) were defined as heritable (43). The sum of relative abundance of every single heritable and nonheritable bacteria was equal to total relative abundance (total proportion).

The microbial contribution to lactation performance, i.e., microbiability, was calculated using ASReml. To capture the relationships among microbiome, their relationship matrix (M) was constructed from O, M was obtained as $M=\frac{1}{q}OO^{T}$ with matrix O of dimension of *n* × *q*, where *n* is the number of animals and *q* is the number of taxa. Each element of the S matrix, S*_ij_* was the relative abundance of taxa *j* in animal *i* (plus 1). The elements of O were calculated as below:

(4) $$O_{\mathrm{ij}}=\frac{\log(S_{\mathrm{ij}})-\bar{\log S_{.j}}}{\mathrm{sd}(\log S_{.j})}$$

Using the microbial taxa, the microbial contribution to lactation performance was calculated using the following model:

(5) $$y^{\text{**}}=1\mu +\mathrm{Uc}+\mathrm{Lm}+e$$

Where y^**^ is lactation traits, including MY, MP, MPY, MF, MFY, ECM, and VFAs, ***μ*** is the overall mean and 1 is a column vector with ones, ***c*** is the fixed effect vector, including the effects of subcohort, parity, age, and days-in-milk, ***U*** is the design matrix; ***m*** is the random microbiome effect following a distribution of **N**(0, **M**σ_*m*^2^_) with the microbiome relationship matrix M and the additive microbiome variance σ_m_^2^, ***L*** is the design matrix; and ***e*** is the random residual effect following **N**(0, **I**σ_*e*^2^_). In ASReml V4.1, the following equation was used to calculate the microbial contribution (12):

(6) $$m^{2}=\sigma_{m^{2}}/(\sigma_{m^{2}}+\sigma_{e^{2}})$$

Because the number of heritable and nonheritable bacteria was different, high microbiability was not equal to high single taxon contribution to traits. Therefore, the average contribution of single taxon to the rumen VFA and lactation trait was estimated using mean microbiability of heritable and nonheritable, respectively. The mean microbiability was referred to in the concept of quantitative genetics. In short, it is assumed that all SNP markers have variance (following standardizing marker genotypes) so the marked genetic variance is given by the sum of individual marker variances. The mean microbiability was defined as follows (51):

(7) $$\beta^{2}=m^{2}/o$$

Where ***β***^2^ is mean microbiability of a single taxon. ***m***^2^ is microbiability that is a population concept and represents the contribution of all the bacteria (heritable and nonheritable bacteria) to lactation performance, and ***o*** is the number of heritable and nonheritable bacterial taxa, respectively.

The microbial taxa were used to estimate h^2^ and microbiability in ASRmel V4.1 with the following command (43): *–alias asreml =/Applications/asreml/release4/bin/asreml.sh –asreml*. In addition, we tested the relationship between G matrix and M matrix based on mantel test in R package vegan (52).

### Genome-wide association studies.

The GWAS analysis was performed based on the calculated h^2^. The bacteria with h^2^ estimation significantly greater than zero ($\frac{h^{2}}{\mathrm{SE}}>2$, *P* < 0.05) were selected (53), and the microbial taxa and genotype of h^2^ estimation were used for GWAS analysis. Microbial taxonomic features were adjusted for the fixed effects and covariate, including age, parity, pH, and days-in-milk in the above model/equation (Equation 1). The phenotype and genotype data after quality control was used in the rrBLUP (54, 55) package in R using the following model:

(8) $$y^{\#}=1\mu +\mathrm{Za}+\mathrm{Jg}+e$$

Where ***y***^#^ is the adjusted values of microbial taxa; ***a*** and ***e*** are the random additive genetic effect and the random residual effect with assumptions of distribution, variance and covariance structure as descripted above, ***Z*** is the design matrix; and ***g*** is a fixed effect modeling the additive SNP effect, ***J*** is the design matrix. Genotypes were coded as −1/0/1 for AA, AB, and BB. For the *P* value calculated for each trait, the Benjamini-Hochberg method (56) was used to justify the *P* value. When *P*_adj_ < 0.01, the SNP-microbial taxa association was regarded as significant; and when 0.01 < *P*_adj_ < 0.05, the association was defined as tentatively significant.

### Network analysis.

Correlation networks were generated to explore the relationship among heritable rumen bacteria, rumen VFAs, and lactation performance; and correlation analysis was performed in R software with Spearman’s correlation method. The correlation coefficients greater than 0.2 and *P*_adj_ < 0.05 or less than −0.2 and *P*_adj_ < 0.05 were considered significant. The correlation network was generated by Spearman’s rank correlation and visualized by Cytoscape v3.8.2.

### Data availability.

The rumen bacteria Illumina sequencing raw data for our samples are from the NCBI Sequence Read Archive (SRA) under accession numbers: PRJNA597489 (7, 57), and PRJNA741384 (48). The variation data reported in this paper have been deposited in the Genome Variation Map (GVM) in China National Center for Bioinformation, under accession numbers PRJCA004923 and are publicly accessible at http://bigd.big.ac.cn/gvm with project number are GVM000134 and GVM000274.
