# Supplementary figures and images for: Heritable and Nonheritable Rumen Bacteria Are Associated with Different Characters of Lactation Performance of Dairy Cows
Source: mSystems. 2022 Sep 14;7(5):e00422-22. doi: 10.1128/msystems.00422-22 (PMC9600476; doi:10.1128/msystems.00422-22)

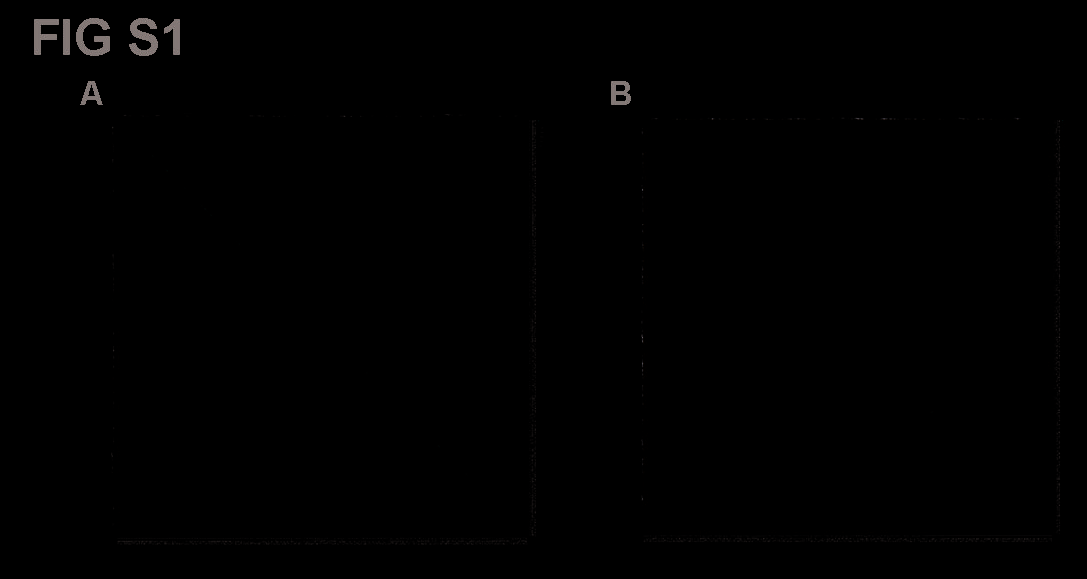

Supplement: FIG S1 [file msystems.00422-22-s0010.tif]
